# PTPRD gene variant rs10739150: A potential game-changer in hypertension diagnosis

**DOI:** 10.1371/journal.pone.0304950

**Published:** 2024-06-27

**Authors:** Laith AL-Eitan

**Affiliations:** 1 Department of Biotechnology and Genetic Engineering, Faculty of Science and Arts, Jordan University of Science and Technology, Irbid, Jordan; 2 Whitehead Institute for Biomedical Research, Massachusetts Institute of Technology, Cambridge, MA, United States of America; PearResearch / Government Doon Medical College, INDIA

## Abstract

**Background:**

High blood pressure, also known as hypertension (HTN), is a complicated disorder that is controlled by a complex network of physiological processes. Untreated hypertension is associated with increased death incidence, rise the need for understanding the genetic basis affecting hypertension susceptibility and development. The current study sought to identify the genetic association between twelve single nucleotide polymorphisms (SNPs) within seven candidate genes (*NOS3*, *NOS1AP*, *REN*, *PLA2G4A*, *TCF7L*, *ADRB1*, and *PTPRD*)

**Methods:**

The current study included 200 Jordanian individuals diagnosed with hypertension, compared to 224 healthy controls. Whole blood samples were drawn from each individual for DNA isolation and genotyping. The SNPStats tool was used to assess haplotype, genotype, and allele frequencies by the mean of chi-square (χ2).

**Results:**

Except for rs10739150 of PTPRD (P = 0.0003), the genotypic and allelic distribution of the SNP was identical between patients and controls. The prevalence of the G/G genotype in healthy controls (45.5%) was lower than in hypertension patients (64.3%), suggesting that it might be a risk factor for the disease. PTPRD TTC genetic haplotypes were strongly linked with hypertension (P = 0.003, OR = 4.03).

**Conclusion:**

This study provides a comprehensive understanding of the involvement of rs10739150 within the *PTPRD* gene in hypertension. This new knowledge could potentially transform the way we approach hypertension diagnosis, providing an accurate diagnostic tool for classifying individuals who are at a higher risk of developing this condition.

## 1. Introduction

Hypertension is one of the leading causes of heart attacks, strokes, and coronary heart disease, and the major risk factor for heart and renal failure. All of which can result in premature mortality, morbidity, and significant economic burdens [1–9]. According to statistics, about 116.4 million people are affected with hypertension, which is found to be associated with 2303 deaths from cardiovascular disease (CVD) per day [4, 8, 10, 11]. The global prevalence of hypertension in the adult population is more than 26%, and it is expected to rise to 30% by 2025, creating a significant public health concern in both developed and developing countries [12]. Taking into consideration that it varies with age, gender, and ethnicity, as well as influenced by dietary habits, such as salt and potassium consumption [13, 14]. Effective hypertension management is critical for preventing related complications such as heart failure, coronary artery disease, atrial fibrillation, and ischemic stroke [15, 16]. Despite guideline-driven treatment, approximately one-third of the individuals achieve target blood pressure [8, 15, 17]. Blood pressure (BP) is a quantitative variable that is normally distributed in the general population, with inherited genetic factors accounting for 30–50% of the variance [8–10, 18–21].

Candidate gene association studies have been conducted to evaluate the impact of SNPs, haplotypes, and allele combinations at multiple polymorphisms on drug response characteristics [8, 9, 20]. In pharmacogenomics, genome-wide association studies (GWAS) identified polymorphisms related to effectiveness and adverse drug responses such as *eNOS*, *ADRB1*, *REN*, and *PTPRD* [8, 9, 19, 22, 23]. The endothelial nitric oxide synthase 3 (eNOS, commonly known as *NOS3* can cause the fundamental release of nitric oxide in endothelial cells, which has Vaso protective effects in controlling BP [20, 24]. The *NOS3* gene has many single nucleotide variants, such as (T-786C) or *rs2070744*. Many studies have found a link between the T-786C allele and an increased risk of coronary artery disease and hypertension [24–26].

Human *ADRB1* is a seven-transmembrane G-protein-coupled receptor that is expressed in cardiac myocytes [14, 19]. Two variants in the *ADRB1* coding area have been identified, *rs1801252 (Ser49Gly*) and *rs1801253 (Arg389Gly)*) [19, 27]. Both mutations are found in the intracellular cytoplasmic tail of the receptor, near the 7^th^ transmembrane region, which is a probable Gs-protein binding domain [19, 27]. Renin, a protease expressed mostly in the kidney, hence catalyses the first and rate-limiting step of the RAS cascade, playing an important role in the control of blood pressure and electrolyte balance [28]. The *REN* gene is made up of 12 exons spread across 12 kb of human chromosome 1q32 [28]. Several SNPs within the *REN* gene have been studied for their relationships with hypertension in diverse ethnic groups, with inconsistent results [26, 28]. While numerous studies have found negative relationships between *REN* polymorphisms and hypertension, favourable connections have also been documented in several ethnic groups including the United Emirates and Spanish populations, as well as Han, Tibetan, Mongolian, Indian, and Caucasian in the USA and a few other populations [28]. The *PTPRD* gene encodes the enzyme receptor-type tyrosine-protein phosphatase delta that belongs to the protein tyrosine phosphatase (PTP) family [29, 30]. PTPs are signalling molecules that govern a wide range of biological activities, including cell proliferation, differentiation, the mitotic cycle, and neoplastic transformation [30, 31]. Multiple gene families have been associated with cardiovascular diseases associated with multiple genetic variants distributed among the Jordanian population. Studies have also shown that multiple SNPs could also be associated with the response to cardiovascular drugs like warfarin [32–34]. In this study, we aim to further investigate the possible association of selected variations (*NOS3 (rs2070744)*, *NOS1AP (rs10494366)*, *REN (rs11240688)*, *PLA2G4A (rs1015710)*, *TCF7L2 (rs4506565*, *rs4132670*, *and rs7917983)*, *ADRB1(rs1801252 and rs1801253)*, and *PTPRD (rs4742610*, *rs10739150*, *and rs12346562)* with hypertension in the Jordanian population and the effect on drug response and clinical symptoms.

## 2. Materials and methods

### 2.1. Study design

According to the World Health Organization (WHO), the global prevalence of hypertension stands at 1.28 billion cases. In Jordan, out of a total population of 10,699,000, 1.3 million adults aged 30–79 years are affected by hypertension, with a prevalence of 12%, as reported by the WHO (https://www.who.int/publications/m/item/hypertension-jor-2023-country-profile). The sample size required to estimate a population proportion (p) of a large population with (1−α)100% confidence and an error no greater than ε can be calculated using the formula:

$$n=\frac{z_{\alpha /2}^{2}\hat{p}(1-\hat{p})}{\epsilon^{2}}$$

For a confidence interval of 95%, Zα/2 is equal to 1.96. Assuming a maximum error rate of 0.05 and a sample proportion of 0.12 (reflecting the prevalence of hypertension in Jordan), the calculated sample size is n = 163. In our study, the sample size for the cases was 200 (initially, we screened 320 patients, but after applying the exclusion criteria, it was reduced to 200), which exceeds the required sample size, thus meeting the necessary criteria.

Based on the result of statistical power calculation, the recruitment for this study started on August 27, 2020 and concluded on April 6, 2023. Blood samples were collected from 224 healthy controls and 200 hypertension patients who are of Jordanian origin and attended a clinic at the Coronary Cardiac Care Department at King Abdullah University Hospital (KAUH), Irbid, Jordan. This study was approved by the Humans Ethics committee of Jordan University of Science and Technology and KAUH under the study number 4/133/2020. The blood samples were collected after the participants signed the informed consent.

The patient cohort examined in our study consisted of individuals who regularly attend hypertension clinics within the Coronary Cardiac Care Department at King Abdullah University Hospital (KAUH). Diagnosis of hypertension was based on the ICD-10 coding system, ensuring inclusion of confirmed hypertensive patients. Considering the consistent attendance of these individuals at hypertension clinics and their ongoing treatment regimen, additional confirmation of the disease was deemed unnecessary. Therefore, our study focused solely on collecting blood samples and clinical records to adhere to the outlined methodology.

Participants were classified as having hypertension if their systolic blood pressure (SBP) exceeded the predefined threshold of 140 millimetres of mercury (mmHg) and their diastolic blood pressure (DBP) exceeded 90 mmHg. The study included 200 patients meeting specific inclusion criteria, which required official confirmation of hypertension diagnosis, data availability in the KAUH patient registry system, age of 35 years or older, diagnosis of high blood pressure from different classes, and prescription of antihypertensive medications. Patients were categorized into five groups based on the type of antihypertensive medication they were prescribed (ACEIs, ARBs, CCBs, BBs, and TDs), adhering to guidelines established by the Joint National Committee on Detection, Evaluation, and Treatment of High Blood Pressure (JNC) and the American Heart Association (AHA), as practiced at KAUH. Exclusion criteria encompassed individuals declining to provide written informed consent, those lacking adequate clinical data, and individuals with familial relationships up to the second degree with current study participants. Additionally, individuals not receiving prescribed antihypertensive medications were deemed ineligible for inclusion. All the demographic data for the patients were taken from the electronic registration system of the hospital and are shown in **Table 1**.

**Table 1:** Sample demographic data.

Category	Subcategory	Percentage (%)/Mean ± SD	
		Controls	HTN Patients
Gender	Male	61.7%	58.4%
	Female	38.3%	41.6%
Age	-----------	34.5088± 12.443	58.8477±10.395
BMI	-----------	30.56±56.26	33.35 ±22.58
Smoker	Yes	44%	27.9%
	No	56%	72.1%

### 2.2. Treatment approach and measures

The prescribed treatment schedule and protocol of each patient was assessed and classified. Firstly, simple interviews were held with the patients to collect demographic information, background about their treatment and its effectiveness. The participants were also asked about their lifestyle and daily activities such as smoking, drinking, work, fitness, and diet. During the interview, the participants were also told to confirm their lab results (CBC and Lipid profile) and any comorbidities like diabetes mellitus, ischemic heart disease, heart failure, peripheral vascular disease, cerebrovascular accident, chronic kidney disease, dialysis, atrial fibrillation. Also, during the interview, the blood pressure for each patient was measured. The data collected from the interviews were compared with those available in the KAUH patient registry system and track record. All the data were collected in a time range of a single year from the start of the treatment protocol.

### 2.3. Extraction of genomic DNA

gDNA was extracted from the collected blood samples using the Qiagen Blood DNA extraction kit following the manufacturer’s instructions. The quantity and quality of the DNA were assessed using NanoDrop and 1000® spectrophotometers and gel electrophoresis.

### 2.4. SNP selection and genotyping

The selection of SNPs was meticulously conducted to encompass a comprehensive array of genetic variations associated with cardiovascular diseases, thereby revealing an intricate network of genes interconnected in their involvement with hypertension. *NOS3* gene encodes endothelial nitric oxide synthase (eNOS), an enzyme crucial for producing nitric oxide (NO) in endothelial cells. NO plays a pivotal role in vasodilation and vascular homeostasis, regulating blood pressure. Variations in the *NOS3* gene, such as the *rs2070744* SNP, have been associated with alterations in NO production and endothelial function, contributing to hypertension and related cardiovascular disorders. The association of NOS3 variants with coronary artery disease, myocardial infarction, and strokes underscores its importance in cardiovascular health and hypertension pathogenesis [25]. *NOS1AP* gene encodes a nitric oxide synthase 1 adaptor protein and regulates the function of neuronal nitric oxide synthase (nNOS) and is involved in cardiac repolarization. Genetic variants in *NOS1AP* gene, such as *rs10494366*, have been associated with prolonged heart rate-corrected intervals and an increased risk of cardiac death. Dysregulation of cardiac repolarization can predispose individuals to arrhythmias and cardiovascular events, including hypertension-related complication [35]. Renin is a key enzyme in the renin-angiotensin-aldosterone system (RAAS), regulating blood pressure and fluid balance. The *rs11240688* SNP in the *REN* gene has been linked to the response to hydrochlorothiazide (HCTZ), a commonly used diuretic for hypertension treatment. Variations in the REN gene may influence renin production and RAAS activity, affecting blood pressure regulation and hypertension susceptibility [10].

*PLA2G4A* gene encodes a member of the phospholipase A2 family involved in lipid metabolism and inflammatory responses. The *rs1015710* SNP in PLA2G4A has been associated with predicting high-density lipoprotein cholesterol levels in individuals treated with atenolol. Dyslipidemia is a common risk factor for hypertension, and genetic variations in PLA2G4A may contribute to lipid abnormalities and hypertension development [10]. TCF7L2 is involved in Wnt signaling and regulates gene expression associated with glucose and lipid metabolism. The *rs4506565* SNP in *TCF7L2* gene has been linked to increased body mass index (BMI) and systolic blood pressure. Genetic variants in *TCF7L2* may influence insulin sensitivity, adiposity, and blood pressure regulation, contributing to hypertension risk [36]. *rs4132670* has been mostly correlated with type 2 diabetes [37] and *rs7917983* has been associated with increased risk of diabetes mellitus [38]. *ADRB1rs1801252 and rs1801253* SNPs were associated with hypertension [19, 39]. In *PTPRD*, *rs4742610* was corelated with resistant hypertension in white and Hispanic people, *rs10739150* was correlated with response to antihypertension medication in black people, while *rs12346562* was associated with response to β-blockers in Finnish people [31]. The selection of SNPs was informed by comprehensive data sources including, National Centre for Biotechnology Information (NCBI) (http://www.ncbi.nlm.nih.gov/SNP/), Ensemble database (http://www.ensembl.org/index.html) and the Applied Biosystems SNP database (http://www.appliedbiosystems.com) databases. To genotype the targeted gene variants, the Sequenom MassARRAY technique was employed at the Australian Genome Research Facility (AGRF; Melbourne Node, Melbourne, Australia) in adherence to the manufacturer’s guidelines. Detailed primer design for each SNP is provided in **S1 Table**, ensuring accurate genotyping and reliable data interpretation.

### 2.5. Statistical analyses

Statistical analyses were conducted using various methods and software tools to explore the relationship between genotype and phenotype, as well as to assess the association of genetic variants with disease status. Pearson’s chi-square test and one-way ANOVA were employed to analyze the genotype-phenotype relationship, while Odds Ratios (OR) with 95% confidence intervals (CI) were calculated. These analyses were performed using the Statistical Package for Social Sciences (SPSS) version 26.0 (SPSS, Inc., Chicago, IL). Post hoc multiple comparison tests were employed to study genotype-per-genotype associations. Multinomial logistic regression analyses, incorporating clinical and demographic covariables, were utilized to identify potential effects of gene polymorphisms on disease. Odds ratios (ORs) were also used to express associations, with a significance threshold set at p < 0.05. The Hardy-Weinberg Equilibrium was assessed using the chi-square test, considering p > 1 × 10−6 as indicative of normal distribution. Multinomial logistic regression was performed using SPSS version 25.0.

Additionally, SNP stat (https://www.snpstats.net/start.htm) was utilized to investigate multiple inheritance models, Hardy-Weinberg Equilibrium, haplotype frequency estimates, and the connection between haplotypes and disease status. P-values below 0.05 were considered statistically significant. To address multiple testing corrections, the effective number of SNPs was determined according to the method proposed by [40]. Additionally, the Bonferroni correction was applied, setting the significance cut-off at α/n, where α = 0.05 and n represents the number of tests [41]. Through these rigorous statistical analyses, we aimed to comprehensively evaluate the genetic factors associated with disease susceptibility and phenotype expression while minimizing the potential for Type I errors.

## 3. Results

### 3.1. Demographic characteristics and Hardy-Weinberg Equilibrium

This study included 224 healthy controls with a mean age of 34.088 years ±12.443, with 61.7% of them being males, compared to 58.4% of the 200 hypertension patients aged 58.8477 years ±10.395. The body mass index (BMI) was obtained for both controls (30.56±56.26) and hypertension patients (33.35 ±22.58) **(Table 1)**. However, there is no considerable variation in the other parameters. **Table 2** shows that the genotype distribution in the study population followed HWE (*P* > 0.05).

**Table 2:** The genes, their SNPs, their minor allele frequencies, and HWE p-value in cases and controls.

Gene	SNP_ID	Control (n = 224)			Cases (n = 200)		
		MA	MAF	HWE p-value	MA	MAF	HWE p-value
*PTRD*	*rs4742610*	T	0.36	0.094	T	0.33	0.42
	*rs10739150*	T	0.32	0.47	T	0.21	0.13
	*rs12346562*	A	0.31	0.47	A	0.36	1
*PLA2G4A*	*rs1015710*	G	0.03	1	G	0.06	0.52
*REN*	*rs11240688*	A	0.21	0.03	A	0.21	0.083
*NOS3*	*rs2070744*	C	0.3	0.72	C	0.32	1
*NOS1AP*	*rs10494366*	G	0.47	0.27	G	0.46	0.56
*TCFL2*	*rs7917983*	T	0.38	1	T	0.37	0.76
	*rs4132670*	A	0.4	0.15	A	0.43	0.15
	*rs4506565*	T	0.36	1	T	0.42	0.19
*ADRB1*	*rs1801252*	G	0.07	0.2	G	0.07	0.25
	*rs1801253*	G	0.48	1	G	0.39	0.3

MA: Minor allele; MAF: Minor allele frequency; HWE: Hardy-Weinberg Equilibrium.

### 3.2. Allele and genotype frequencies and genetic model analysis for the association of genetic polymorphism with hypertension

**Tables 3 and 4**, as well as **S2 Table** illustrate genotype and allele frequency distributions and genotype models of the examined polymorphisms across the study groups showing no significant association, except for *rs10739150* of *PTPRD* (*P* = 0.0003), *rs1015710* of *PLA2G4A* (*P* = 0.044), *rs4506565* of *TCFL2* (*P* = 0.038) and *rs1801253* of *ADRB1* (*P* = 0.017). There is a considerable difference in the frequency of the G/G genotype in healthy controls (45%) against hypertension patients (64%) at *rs10739150* SNP, which might be a potential risk factor for the disease **(S2 Table)**. The genetic model study found that the codominant model of the *rs10739150* SNPs (OR = 2.20, *P* = 0.0015), the dominant model (OR = 2.16, *P* = 0.0003), and the over dominant model (OR = 2.03, *P* = 0.0012) were significantly associated with hypertension **(S2 Table)**. **Table 3** shows the genotype distribution of *PTPRD rs10739150* across both patients and controls. In that, the dominant G/G genotype was found in 64% of the controls, and 45% of the patients. The G/T genotype was found in 30% of controls and 46% of the patients. The T/T genotype was less commonly found in both controls and patients (6% and 8% respectively). Thus, concluding that *rs10739150* is significantly associated with onset of hypertension (*P =* 0.0003). Another significant association was found with the dominant model of *rs1801253* of *ADRB1* (OR = 1.68, *P* = 0.021, **S2 Table**). *TCF7L2 rs4506565* has showed that the recessive model showed significant association *(P =* 0.042, **S2 Table**) with the development of hypertension.

**Table 3:** Genotype distributions of SNPs within genes in hypertension patients and control.

Gene	SNP_ID	Genotype	Frequency		P-value	Overall P-value
			Controls	Cases		
*PTRD*	*rs4742610*	C/C	65 (38%)	85 (43%)	0.184	0.33
		C/T	88 (52%)	93 (47%)	0.180	
		T/T	16 (9%)	19 (10%)	0.911	
	*rs10739150*	G/G	75 (45%)	126 (64%)	0.001	**0.0003**
		G/T	76 (46%)	58 (29%)	0.003	
		T/T	14 (8%)	12 (%6)	0.012	
	*rs12346562*	C/C	76 (46%)	80 (41%)	0.158	0.147
		C/A	76 (46%)	92 (47%)	0.871	
		A/A	14 (8%)	25 (13%)	0.076	
*PLA2G4A*	*rs1015710*	A/A	159 (0.94)	174 (0.88)	0.025	**0.044**
		G/A	10 (0.06)	22 (0.11)	0.035	
		G/G	0 (0)	1 (0.01)	0.184	
*REN*	*rs11240688*	A/A	12 (7%)	13 (7%)	0.676	0.63
		A/G	45 (27%)	57 (29%)	0.344	
		G/G	111 (66%)	127 (64%)	0.561	
*NOS3*	*rs2070744*	T/T	92 (47%)	81 (48%)	0.841	0.53
		C/T	85 (43%)	74 (44%)	0.841	
		C/C	20 (10%)	14 (8%)	0.308	
*NOS1AP*	*rs10494366*	T/T	49 (30%)	59 (30%)	0.664	0.62
		G/T	73 (45%)	92 (47%)	0.994	
		G/G	39 (24%)	43 (22%)	0.774	
*TCFL2*	*rs7917983*	C/C	66 (39%)	79 (40%)	0.841	0.82
		T/C	79 (47%)	90 (46%)	0.893	
		T/T	24 (14%)	28 (14%)	0.944	
	*rs4132670*	G/G	65 (38%)	69 (35%)	0.274	0.44
		G/A	72 (43%)	86 (44%)	0.921	
		A/A	32 (19%)	42 (21%)	0.304	
	*rs4506565*	A/A	70 (41%)	70 (36%)	0.076	**0.038**
		A/T	78 (46%)	87 (44%)	0.340	
		T/T	21 (12%)	40 (20%)	0.045	
*ADRB1*	*rs1801252*	A/A	144 (87%)	171 (87%)	0.921	0.65
		A/G	20 (12%)	24 (12%)	0.902	
		G/G	2 (1%)	2 (1%)	0.814	
	*rs1801253*	C/C	46 ((27%	76 (39%)	0.017	**0.017**
		C/G	84 (50%)	87 (44%)	0.271	
		G/G	39 (23%)	34 (17%)	0.161	

P-values< 0.0042 (0.05/# of SNPs, 0.05/12 = 0.0042 after applying multiple comparison) are considered as significant. NA: not available.

**Table 4:** Allele distributions of SNPs within genes in hypertension patients and control.

Gene	SNP_ID	Allele	Frequency		P-value
			Control	Case	
*PTRD*	*rs4742610*	C	218 (64.5%)	263 (66.8%)	0.52
		T	120 (35.5%)	131 (33.2%)	
	*rs10739150*	G	226 (68.5%)	310 (79%)	**0.0012**
		T	104 (31.5)	82 (21%)	
	*rs12346562*	C	228 (68.7)	252 (63.9)	0.181
		A	104 (31.3)	142 (36.1)	
*PLA2G4A*	*rs1015710*	A	328 (97%)	370 (93.9)	**0.044**
		G	10 (3%)	24 (6.1%)	
*REN*	*rs11240688*	A	69 (20.5%)	83 (21.1%)	0.86
		G	267 (79.5%)	311 (78.9%)	
*NOS3*	*rs2070744*	T	269 (68.3%)	236 (69.8%)	0.65
		C	125 (31.7%)	102 (30.2%)	
*NOS1AP*	*rs10494366*	T	171 (53.1%)	210 (54.1%)	0.78
		G	151 (46.9%)	178 (45.9%)	
*TCFL2*	*rs7917983*	C	211 (62.4%)	248 (62.9%)	0.88
		T	127 (37.6)	146 (37.1%)	
	*rs4132670*	G	202 (59.8%)	224 (56.9%)	0.42
		A	136 (40.2%)	170 (43.1%)	
	*rs4506565*	A	218 (64.5%)	227 (57.6%)	0.057
		T	120 (35.5%)	167 (42.4%)	
*ADRB1*	*rs1801252*	A	308 (92.8%)	366 (92.9)	0.94
		G	24 (7.2%)	28 (7.1%)	
	*rs1801253*	A	176 (52.1%)	239 (60.7%)	**0.019**
		G	162 (47.9%)	155 (39.3%)	

P-values< 0.0042 (0.05/# of SNPs, 0.05/12 = 0.0042 after applying multiple comparison) are considered as significant. NA: not available.

### 3.3. Haplotype analysis of ADRB1, PTRD, and TCF7L2 gene variants

The haplotype analysis as shown in **Table 5**, reveals that the AG haplotype of *ADRB1* (*P* = 0.017, OR = 1.44), the TTC of *PTPRD* (*P* = 0.0033, OR = 4.03), and the CAA of *TCF7L2* (*P* = 0.012, OR = 6.79) are significantly associated with hypertension and may be considered protective factors.

**Table 5:** Haplotype analysis of ADRB1, PTRD, TCF7L2 gene variants among hypertension patients and healthy controls.

Gene	Haplotypes	Frequency		Odd ratio (95%)	P-value
		Controls	Cases		
*PTRD*	CGC	0.2626	0.3334	1	N/A
	TGC	0.1926	0.1736	1.29 (0.78–2.15)	0.32
	CGA	0.173	0.1891	1.10 (0.64–1.91)	0.72
	CTC	0.1254	0.0957	1.50 (0.75–3.02)	0.25
	TGA	0.0561	0.0946	0.64 (0.28–1.46)	0.29
	CTA	0.0839	0.0493	1.92 (0.77–4.77)	0.16
	TTC	0.1064	0.0368	4.03 (1.60–10.14)	**0.0033**
	TTA	0	0.0274	0.00 (-Inf—Inf)	1
*TCF7L2*	CAT	0.269	0.334	1	N/A
	TGA	0.286	0.281	1.24 (0.85–1.81)	0.27
	CGA	0.2959	0.2694	1.33 (0.88–2.01)	0.17
	TAT	0.0703	0.0717	1.17 (0.56–2.43)	0.68
	CAA	0.0489	0.0079	6.79 (1.54–29.92)	**0.012**
	TAA	0.0142	0.0179	1.13 (0.27–4.82)	0.87
	CGT	0 .0105	0.0182	0.73 (0.17–3.04)	0.66
*ADRB1*	AC	0.4472	0.5355	1	N/A
	AG	0.4793	0.3934	1.44 (1.07–1.95)	**0.017**
	GC	0.0735	0.0711	1.25 (0.71–2.20)	0.45
	GG	0	0	N/A	N/A

P-values< 0.0042 (0.05/# of SNPs, 0.05/12 = 0.0042 after applying multiple comparison) are considered as significant. NA: not available.

### 3.4. Genotype-phenotype association analysis among hypertension patients

Genotype-phenotype association analysis in **S3 Table**, shows that *ADRB1 rs1801253* exhibited noteworthy association with peripheral vascular disease (*P* = 0.001), angiotensin converting enzyme inhibitors (ACEi) (*P* = 0.005*)* and other medication (*P* = 0.027). The *rs1801252* is associated with cerebrovascular accident (*P* = 0.022). *REN rs11240688* was significantly connected with Stomach medications (*P* = 0.013). *PTPRD rs10739150* meaningfully associated with creatinine (*P* = 0.003) and dialysis (*P* = 0.048), meanwhile *rs4742610* with urea (*P* = 0.008) and K level (*P* = 0.002). *TCF7L2 rs7917983* has a significant relationship with antiplatelet (*P* = 0.015). *PLA2G4A rs1015710* is associated with ischemic heart disease (*P* = 0.013). For the *NOS1AP rs10494366* is significantly linked with diet (*P* = 0.008).

### 3.5. Post hoc and regression analysis

In this study, we employed regression analysis as a powerful statistical tool to delve into the intricate relationships between various key features observed within both the case and control groups. Regression analysis allowed us to investigate how hypertension, represented by both systolic and diastolic blood pressure measurements, correlates with different clinical characteristics among the cases. By examining these associations, we aimed to uncover potential factors contributing to the development and progression of hypertension in our study population. Furthermore, our analysis extended beyond merely exploring the relationship between hypertension and clinical features. We also sought to elucidate any potential connections between the observed clinical characteristics within the cases and the specific genetic variations represented by the selected SNPs. This aspect of the analysis enabled us to explore the genetic underpinnings of hypertension and to identify any potential genetic markers associated with specific clinical manifestations of the condition.

The detailed outcomes of the regression analysis, encapsulating the complex interplay between hypertension, clinical features, and genetic variations, are presented comprehensively in **S4–S6 Tables**. These tables provide a detailed breakdown of the statistical findings, offering valuable insights into the magnitude and directionality of the relationships observed in our study cohort. Moreover, to further elucidate the significance of our findings, we conducted post hoc tests to compare individuals with different genotypes in terms of clinical symptoms within the case group. These post hoc tests allowed us to identify statistically significant mean differences in variables among the cases, shedding light on potential genotype-phenotype associations and providing further context to our regression analysis results. The findings presented in **S7 Table** serve as a crucial supplement to our regression analysis, offering additional insights into the clinical implications of genetic variations associated with hypertension. Overall, through these rigorous analytical approaches, we aimed to comprehensively explore the multifaceted nature of hypertension, unravelling the intricate interplay between genetic factors, clinical characteristics, and the development of this prevalent and complex cardiovascular condition.

## 4. Discussion

Hypertension is a systemic condition characterized by elevated arterial blood pressure and is the leading cause of death from cardiovascular disease [15, 32]. Due to individual vulnerability, hypertension treatment is a challenging aspect in medical care. Genetic variations found to have a significant role in the aetiology of hypertension and its treatment response [24, 42]. Here, we assessed genotypic and allelic frequencies of several SNPs within different candidate genes: *NOS3 (rs2070744)*, *NOS1AP (rs10494366)*, *REN (rs11240688)*, *PLA2G4A (rs1015710)*, *TCF7L2 (rs4506565*, *rs4132670*, and *rs7917983)*, *ADRB1 (rs1801252* and *rs1801253)*, and *PTPRD (rs4742610*, *rs10739150*, and *rs12346562)* and their association with hypertension in Jordanian community.

The r*s12346562* SNP is identified on chromosome 9p23 upstream of the *PTPRD* gene, whereas the *rs1073915*0 is found downstream of *PTPRD* [31]. This gene encodes the protein tyrosine phosphatase receptor type D, a member of the highly conserved family of receptor protein tyrosine phosphatases [31]. Although *rs12346562* negatively affects the response to hypertension therapy in white participants, it is not associated with treatment response in black hypertensive individuals [31]. According to current findings, *PTPRD rs10739150* is the only SNP that might be a risk factor for hypertension, since there is a significant difference in the frequency of the G/G genotype in healthy controls (45.5%) compared to the hypertensive group (64.3%), in which G allele shows a higher rate within patients.

*NOS3* generates nitric oxide (NO), which is essential for the modulation of vascular tone [32]. A previous study has revealed that the *rs2070744* mutation results in the down regulation of the gene, which can cause coronary spasms [32]. Several investigations revealed a persistent difference in the distribution of *NOS3* variation in black vs white individuals, and this interethnic difference was not altered by geographic origin [42]. Gamil et al. also discovered that participants with genotype C of *rs2070744* had greater systolic pressure and were more likely to develop hypertension [42]. On the other hand, Kui et al. observed that *rs2070744* had no effect on hypertension rates in Japanese and Caucasian populations [43], which is consistent with our findings. In Chinese, Tibetan, Canadian, and Tunisian populations, hypertensive carriers of the CC genotype of *rs2070744* who were treated with anti-hypertensive medicines, had an increased risk of treatment resistance compared to patients with the CT or TT genotypes [22, 24, 25]. The *rs2070744* mutation decreases eNOS protein production, causing NO levels to fall while blood pressure rises, which might explain the mechanism behind the *rs2070744* mutation and hypertension development [32, 33]. Moreover, it has been demonstrated that the *rs2070744* variant alone does not contribute to hypertension unless coupled with other variants [24, 43].

The adrenergic receptors 1 (ADRB1) are targets for many endogenous and pharmacological signalling molecules, including epinephrine, norepinephrine, and β-blocker medications [44]. A nonsynonymous variation of *ADRB1* (*rs1801253*, Arg389Gly) is found to modify blood pressure responsiveness to β-blocker medication in various studies, as it impacts the treatment outcomes of the European population [44]. Several genetic association studies revealed that the Arg allele of the *ADRB1 rs1801253* is related to hypertension risk, but the Gly allele confers a decreased risk in Asian and Chinese populations [19, 27]. Furthermore, there is a definite link between this polymorphism and antihypertension response to β-blockers, such as metoprolol and carvedilol [27]. According to Karlsson and colleagues, the *rs1801252* (Ser49Gly) ADRB1 polymorphism has no impact in hypertensive patients, and the prevalence of the Arg389Gly and Ser49Gly polymorphisms is not different between patient and control groups of Chinese people [19], which also supports our result in Jordanian population. Studies on the Jordanian population have shown varying association between different cardiovascular diseases related genes [45, 46]. Those studies, aligning with others, have also shown that genetic variants could affect the patients’ response to cardiovascular drugs such as warfarin [47, 48]. Therefore, coinciding with the goals of the current study of providing further evidence for possibly treating cardiovascular diseases like hypertension in a pharmacogenomic manner [49, 50].

*REN* SNP lacks association with hypertension in this study, as well as in other studies conducted in Japanese and Malaysian populations [51]. Earlier research in Chinese, Dutch, Arab, and American populations found a link between the *REN* (10607 G>A) polymorphism and hypertension, in addition to other diseases, such as stroke and left ventricular hypertrophy, although results were contradictory [51]. The lack of association between the majority of the studied variants with hypertension seen in this study among hypertensive Jordanians contradicts earlier studies in several different populations, which might be explained by several factors [19, 51]. First, the frequencies of alleles, genotypes, haplotypes, and linkage disequilibrium (LD) patterns may differ amongst ethnic groups [19]. Second, the varying frequency and clinical features of hypertension in various ethnic communities may have an impact on the study’s findings [19, 27]. Third, several techniques of sample selection, as well as inclusion and exclusion criteria, were applied [31, 34]. Furthermore, the intricate relationships between the examined genes and other relevant genes may impact the aetiology of hypertension and the therapeutic response [27].

In order to gain deeper insights into the association between genetic polymorphisms and the heightened susceptibility to hypertension, as well as the responsiveness to treatment, forthcoming investigations should delve into an analysis of patients’ lifestyles and habits. Such an exploration is crucial, as lifestyle factors may interact with genetic biomarkers, potentially influencing the efficacy of treatment approaches. This integration of genetic and lifestyle data holds promise for the advancement of personalized medicine tailored specifically for hypertension management.

This study endeavours to contribute to this growing body of knowledge by providing valuable insights into the status of hypertension among Jordanian patients from the standpoint of genetic polymorphisms. By shedding light on the genetic factors potentially influencing hypertension risk and treatment response within the Jordanian population, this research aims to pave the way for more targeted and effective therapeutic interventions. Through this holistic approach, encompassing both genetic and lifestyle considerations, we aspire to advance our understanding of hypertension and enhance the prospects for personalized patient care in the realm of cardiovascular health.

## 5. Conclusion

In conclusion, our data imply that only the rs10739150 genotype of the PTPRD gene shows an increased risk of hypertension, and the G allele is strongly related to the disease development. Considering the paucity of studies concerning pharmacogenetics and personalized medicine in Jordan [52–54], larger sample sizes and family-based analyses are needed to understand the biochemical processes and mechanisms by which this genetic variation impacts hypertension susceptibility, as well as to assess the implications on drug responsiveness. Our study is the first of its kind that uses Jordanian Arab descendants, thus raising the question of the effect of genetic polymorphisms and ethnicity on hypertension risk. This would add to the knowledge base towards the development of personalized medicine for hypertension. This underscores the novelty and significance of our research within the context of hypertension studies in Jordan, highlighting the importance of expanding scientific inquiry into understudied ethnic groups to gain a comprehensive understanding of disease risk factors and mechanisms. Finally, by conducting genetic association studies across diverse ethnicities, we aim to strengthen and validate our findings in various population groups. This approach is crucial for corroborating the observed associations and elucidating the broader implications of genetic factors in hypertension susceptibility. As such, our study not only contributes to the understanding of hypertension within the Jordanian context but also lays the groundwork for broader insights into the genetic underpinnings of this complex condition across different ethnic backgrounds.

## Supporting information

S1 Table Primer selection for each SNP. (XLSX)

S2 Table Genetic models and distributions of SNPs within genes in hypertension patients and control. (XLSX)

S3 Table Association between different SNP*s* of the studied genes and the clinical characteristics of hypertension patients’ group. (XLSX)

S4 Table Regression analysis of the association between hypertension (sytosolic and diastolic) and clinical features. (XLSX)

S5 Table Regression analysis of the differences regarding demographic data, risk factors, and genetic variants between the case and control groups. (XLSX)

S6 Table Regression analysis of the differences regarding genetic variants and clinical features within the case group. (XLSX)

S7 Table Post hoc analysis of interactions between genetic variants and the clinical features of the case group. (XLSX)
